# Unpacking Genomic Biomarkers for Programmed Cell Death Receptor-1 Immunotherapy Success in Non–Small Cell Lung Cancer Using Deep Neural Networks: Quantitative Study

**DOI:** 10.2196/70553

**Published:** 2026-01-13

**Authors:** Rayan Mubarak, Fahim Islam Anik, Jean T Rodriguez, Nazmus Sakib, Mohammad A Rahman

**Affiliations:** 1 Cypress Bay High School, Weston, FL, United States; 2 Department of Mechanical Engineering, Khulna University of Engineering and Technology, Khulna, Bangladesh; 3 School of Computing and Information Sciences, Florida International University, Miami, FL, United States; 4 Department of Information Technology, Kennesaw State University, Atrium Building J3218, 1100 South Marietta Pkwy SE, Marietta, GA, 30067, United States, 1 4147975981

**Keywords:** lung cancer, machine learning, deep neural network, DeepImmunoGene, biomarkers, RNA-seq analysis, differential gene expression, programmed cell death receptor-1, immunotherapy

## Abstract

**Background:**

Non–small cell lung cancer (NSCLC) is one of the leading causes of cancer-related mortality. Programmed cell death receptor-1 (PD-1) immunotherapy has shown results in the treatment of NSCLC; however, not all patients respond effectively to it. Identifying predictive biomarkers for PD-1 therapy response is critical to improving patient outcomes and treatment strategies. Traditional methods of biomarker discovery often fall short in terms of accuracy and comprehensiveness. Recent advancements in deep learning provide a powerful approach to analyze complex genomic data to resolve this issue.

**Objective:**

This study aims to leverage deep neural networks (DNNs) to identify genomic biomarkers predictive of patient responses to PD-1 immunotherapy in NSCLC. DeepImmunoGene is a model designed using a reduced feature set to identify the most critical biomarkers. We use feature selection to reduce the space and apply deep learning to identify the highly predictive gene subset.

**Methods:**

Differentially expressed genes were identified in RNA-seq data from 355 patients with NSCLC using the LIMMA package in R, followed by preprocessing with log2 transformation, removing outliers, and detecting easily identified genes. Machine learning models, including support vector machines, extreme gradient boosting (XGBoost), and DNNs, were applied to gene expression data to predict patient responses to immunotherapy. Key predictive genes were identified through model interpretation techniques, and differences in model performance were assessed for statistical significance. Primarily, the metric used identifies which genes serve as key biomarkers in regard to immunotherapy detection.

**Results:**

Initially, we identified 1093 differentially expressed genes from RNA-seq data of 355 patients. We then trained models using SVM, XGBoost, and DNN to predict immunotherapy response. The DNN model outperformed both SVM and XGBoost with an accuracy of 82%, an area under the curve of 90%, and recall of 85%. To identify key biomarkers, we performed a permutation importance analysis, narrowing down the gene set to 98 genes. DeepImmunoGene, trained on these 98 genes, showed superior results, with an accuracy of 87% and an area under the curve of 95%. The top 36 upregulated genes in responders and 62 upregulated genes in nonresponders were identified, which could serve as potential biomarkers for predicting response to PD-1 inhibitors. These findings suggest that DeepImmunoGene can reliably forecast immunotherapy outcomes and aid in biomarker discovery, supporting the development of more personalized treatment strategies in NSCLC.

**Conclusions:**

The DeepImmunoGene predictive model identified 36 upregulated genes that may represent candidate genomic biomarkers associated with response to PD-1 immunotherapy in patients with NSCLC. Notably, the 10 most significant genes offer valuable insights into the underlying mechanisms of treatment responses. These biomarkers may not only aid in predicting which patients are more likely to respond to PD-1 immunotherapy but also offer insights into the molecular differences associated with nonresponse.

## Introduction

Lung cancer is a leading cause of cancer-related deaths globally, with approximately 238,340 new cases and 127,070 deaths annually in the United States [1,2] and 2.5 million new cases and 1.8 million deaths worldwide [3]. Smoking accounts for approximately 90% of lung cancer cases [4], whereas the remaining cases in nonsmokers are due to other factors, including environmental exposure to asbestos, arsenic, nickel, pesticides, other toxic chemicals, and air pollution [5,6]. Lung cancer is classified into 2 main groups: small cell lung cancer (SCLC) and non–small cell lung cancer (NSCLC) [4]. SCLC is a rare, fast-growing form of lung cancer that primarily develops in individuals with a long history of tobacco smoking, whereas NSCLC is more common, accounting for 85% of lung cancer cases compared to 15% for SCLC [5]. Although tobacco smoking is a major risk factor for NSCLC, it can also develop in nonsmokers. NSCLC is divided into 3 main types: adenocarcinoma, squamous cell carcinoma, and large cell carcinoma [5,6]. Among these, adenocarcinoma is the most prevalent type, typically developing in the outer parts of the lung and being more common in individuals aged <45 years [5,6]. In contrast, squamous cell carcinoma originates from the epithelial cells of the central airways and is strongly associated with smoking [7,8].

Over the last 10 years, lung cancer treatment has undergone significant changes, with advancements in understanding its biology leading to the development of immunotherapy, which has emerged as a promising therapeutic option [9,10]. Immunotherapy works by enhancing the immune system through the use of drugs that block inhibitory signaling pathways, allowing it to better recognize and eliminate cancer cells [9,10]. Cancer can evade immunosurveillance by expressing ligands for inhibitory checkpoint molecules, such as programmed cell death receptor-1 (PD-1) and cytotoxic T-lymphocyte–associated protein-4, which prevent T cells from recognizing and destroying cancer cells [11]. Thus, immune checkpoint inhibitors (ICIs) have become an effective cancer therapy [12]. In recent years, ICIs have been used as the first line of treatment for metastatic NSCLC as well as consolidation therapy after surgical removal and chemotherapy [10]. PD-1 is a surface receptor found on T cells in lung cancer that acts as a negative regulator of immune responses [13-15]. Recent studies have shown that inhibiting PD-1 or programmed cell death-ligand 1 (PD-L1) restores T cell function, enabling the immune system to recognize and destroy cancer cells, suggesting their potential as promising therapeutic targets for NSCLC treatment [15-17]. However, only a fraction of patients respond to this immunotherapy. Therefore, we aimed to investigate genomic features that may help distinguish responders from nonresponders to PD-1 inhibitors and to gain insight into potential underlying biological differences. Furthermore, researchers have increasingly turned to bioinformatics and machine learning (ML) techniques to discover more precise biomarkers by analyzing large-scale genomic and molecular data. Among ML techniques, deep neural networks (DNNs) are particularly well suited for these tasks due to their ability to process and analyze vast, high-dimensional datasets. The use of ML in this research is indispensable for tackling the complexity of RNA-seq data and addressing the limitations of traditional analytical methods. Traditional statistical methods, such as ANOVA and *t* tests, rely on assumptions such as a normal distribution of the data, which is generally violated in gene expression data. Furthermore, as sample sizes and feature dimensions expand, these approaches also face computational constraints. In contrast, deep learning (DL) methods are particularly well suited to capturing the complex patterns present in genomic data [18]. Such models enable the identification of high-impact biomarkers, uncover nonlinear relationships in gene expression, and generate robust predictions for patient responses to PD-1 immunotherapy.

Several DL approaches have previously been proposed to predict immunotherapy outcomes, including survival-focused models such as DeepSurv and attention-based architectures designed to capture complex transcriptomic interactions [19-23]. These models demonstrate the growing interest in applying advanced DL to immunogenomics. We build upon this foundation by integrating interpretability into our approach. Furthermore, other existing approaches typically rely heavily on imaging-based methods, which can suffer from scanner or protocol heterogeneity and spurious correlation, among others. This study highlights the potential of ML techniques, particularly DNNs, in advancing precision medicine for patients with NSCLC undergoing PD-1 immunotherapy. We applied permutation importance in conjunction with DeepImmunoGene, which identified 98 important genes from a large RNA-seq dataset of 19,911 genes in the Gene Expression Omnibus (GEO) Repository [24]. We trained the DeepImmunoGene model on these genes, which outperformed linear models, achieving an accuracy of 87% and an area under the receiver operating characteristic curve (AUC) of 95%. This model identified a set of 36 upregulated genes in patients with NSCLC who are responders, which may serve as potential biomarkers for predicting responses to PD-1 immunotherapy for this group. Additionally, it identified another set of 62 upregulated genes in patients with NSCLC who are nonresponders, which could act as potential biomarkers for developing ICI therapy for this subgroup. These findings not only offer a foundation for improving patient stratification but also provide insights for tailoring therapeutic strategies. Despite significant advancements in treatment over the past decade, including the development of immunotherapy as a promising strategy for NSCLC, the prognosis for many patients remains poor [25,26]. Although ICIs targeting PD-1 and PD-L1 have shown potential as immunotherapy for patients with NSCLC, only a small fraction of patients respond to PD-1 inhibitors [24].

This underscores the need for more reliable biomarkers to accurately identify patients who will benefit from PD-1 inhibitors. The core work tries to answer 2 research questions (RQs) as follows:

- RQ1: How do ML models perform in predicting patient response to PD-1 immunotherapy based on differentially expressed genes (DEGs)?
- RQ2: What are the key biomarkers identified through feature selection and DL that predict patient response to PD-1 immunotherapy, and how do they contribute to model performance?

## Methods

### Overview

**Figure 1.**
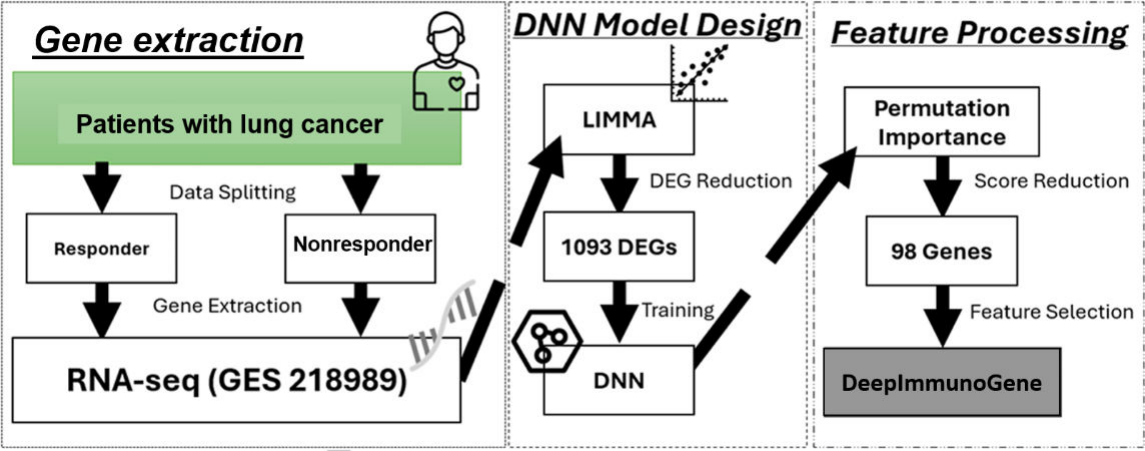
Workflow for identifying biomarkers and predicting programmed cell death receptor-1 immunotherapy response in non–small cell lung cancer. DEG: differentially expressed gene; DNN: deep neural network; SVM: support vector machine; XGBoost: extreme gradient boosting.

The study was carried out according to the workflow presented in Figure 1. This workflow delineates the steps, beginning with the identification of significant DEGs from RNA-seq data [27] using the LIMMA package and culminating in the application of the DeepImmunoGene framework to identify and validate key genes associated with the response to PD-1 immunotherapy in patients with NSCLC.

### Data Acquisition and Preprocessing

We used one RNA-Seq dataset (GSE218989) from the GEO public database GEO Repository [24]. This dataset included gene expression data for 19,911 genes across 355 patients with lung cancer who were treated with either PD-1 or PD-L1 inhibitors. It consisted of 187 nonresponders and 168 responders. Responsiveness was determined by Kang et al [24] using Response Evaluation Criteria in Solid Tumors (RECIST; version 1.1) [28]. Progression-free survival [29] was measured from the start of PD-1/PD-L1 inhibitor therapy to either documented disease progression or death from any cause. Overall survival was measured from the start of PD-1/PD-L1 inhibitor therapy to death from any cause [24]. A responder is therefore classified as a patient who showed improvement under the RECIST criteria or, in other words, a patient who experienced improvements after the PD-1 immunotherapy was administered. At the same time, a nonresponder is a patient who did not meet the criteria showcased by a worsening or stable disease.

The raw gene expression count data were already normalized in the transcripts per million (TPM) value for the 19,911 protein-coding genes. We first identified the DEGs between the responders and nonresponders using the LIMMA package [30] in R (version 4.4.1; Bioconductor, USA). LIMMA was used to create a linear function to model the entire dataset and to develop correlations with response status as the main variable in the design matrix. Empirical Bayes moderation was performed to model and stabilize the gene-wise variances using a prior marginal distribution of the data [30]. Genes with a LIMMA-calculated *P* value less than .05 were considered significantly differentially expressed and were selected for all subsequent analyses and modeling. For model training and testing, the data were further processed by performing a log2 (TPM+1) transformation on each gene expression value to stabilize the variance in gene expression.

### ML Models

#### Overview

The application of ML is vital in this research due to the complexity, scale, and dimensionality of RNA-seq data, as well as the intricate, nonlinear biological mechanisms underlying immunotherapy response in patients with NSCLC [31]. Traditional statistical methods struggle with high-dimensional datasets, such as the 19,911-gene RNA-seq data used here, often succumbing to the “curse of dimensionality” and failing to capture subtle gene interactions. ML models such as support vector machines (SVMs) [32], extreme gradient boosting (XGBoost) [33], and DNN [34] overcome these challenges by effectively handling high-dimensional inputs, modeling complex nonlinear relationships, and identifying important gene features through built-in feature selection techniques. This enables the discovery of meaningful gene patterns that differentiate responders from nonresponders while enhancing predictive power and model generalizability.

Moreover, ML methods excel in managing noise and variability inherent in biological data, offering robust performance through techniques such as regularization and early stopping [35,36]. Their scalability and automation allow for efficient analysis of massive RNA-seq datasets, ensuring accuracy and rapid processing, essential for clinical translation. By integrating advanced techniques for hyperparameter tuning, ML provides a unified, systematic workflow that optimizes predictive performance [37]. These capabilities facilitate the identification of potential predictive biomarkers from gene expression data, which may serve as a foundation for future precision medicine efforts aimed at tailoring immunotherapy strategies in patients with NSCLC. This study used several ML models, including SVM, XGBoost, and DNN [11]. Their predictive performance was evaluated to identify the model that worked best. We built the SVM model using the Python package Scikit-learn (sklearn); for XGBoost, we used the XGBoost Python package [38]; and for the DNN, we used the Keras and TensorFlow Python packages [11]. The details about each ML approach are further described below.

#### Support Vector Machine

SVM is a kernel-based binary classifier that separates key data features linearly into 2 groups in a high-dimensional space called the feature space [38,39]. It searches for the optimal decision boundary (hyperplane) to separate the features by maximizing the margin between the hyperplane and the nearest training data. SVM effectively extracts key but subtle patterns in a complex dataset, allowing for low-error, high-precision sample classification [40]. The model architecture’s hyperparameter settings are given in Table 1.

**Table 1.** Summary of model architectures’ hyperparameter settings.

Model	Key hyperparameters tuned	Final settings	Optimization approach
SVM^a^	C, kernel, gamma	C=0.1, kernel=linear, gamma=0.1	GridSearchCV (5-fold CV^b^)
XGBoost^c^	n_estimators, max_depth, learning_rate, sampling	n_estimators=300, max_depth=100, learning_rate=0.1, sampling=uniform	GridSearchCV (5-fold CV)
DNN^d^	batch_size, epochs, initializer, optimizer, activation, dropout, layers, nodes	Input=256; hidden layers=[128, 100, 100]; activation=ELU^e^; optimizer=Adam; dropout=0; epochs=100; batch size=100	Multistage GridSearchCV

a SVM: support vector machine.

b CV: cross-validation.

c XGBoost: extreme gradient boosting.

d DNN: deep neural network.

e ELU: exponential linear unit.

#### XGBoost 

XGBoost is an ensemble learning algorithm that builds gradient-boosted decision trees one by one and passes the residuals of the previous tree to train the following model. It uses the second partial derivative of the loss function and adds an L1 and L2 regularization term to reduce overfitting [41]. Similar to SVM, we optimized the hyperparameters using GridSearchCV to evaluate a combination of parameters. The hyperparameter settings are given in Table 1.

#### Deep Neural Network

DNN is a nonlinear model that combines neurons that simulate the human brain to make predictions [41,42]. It consists of 3 layers: the input layer, hidden layers, and output layer, which are linked by weights to allow the model to understand complex patterns in the data. We used a DNN because they have been previously applied for genomic-based predictions for diseases [43]. Similar to the previous 2 models, we started with hyperparameter optimization using GridSearchCV. As the DNN has more parameters to tune, we split the Grid Search into 3 stages: (1) batch size and epoch; (2) weight initializer, optimizer, and activation function; and (3) hidden layers, nodes per hidden layer, and dropout optimization. The resulting network consisted of an input layer with 256 nodes, 3 hidden layers with 128 nodes, 100 nodes, and 100 nodes, respectively, an exponential linear unit activation function, Adam optimizer, zero dropout, and normal initializer. The details are summarized in Table 1. We applied the binary cross-entropy loss function as shown in Equation 1 so that the model minimizes to learn the optimal weights for each gene to classify responder and nonresponder patients.


(1) $$L_{BCE}=-\frac{1}{N}\sum_{i=1}^{N}y_{i}\times \log\left(p\left(y_{i}\right)\right)+\left(1-y_{i}\right)\times \log\left(1-p\left(y_{i}\right)\right)$$

The model was trained for 100 epochs with a batch size of 100 based on the GridSearchCV results. After identifying these optimal hyperparameters for the DNN, we used it to construct the architecture for the DeepImmunoGene network.

#### Permutation Importance

To develop the DeepImmunoGene framework, we used the permutation importance method from scikit-learn to identify the subset of genes that most significantly contributed to the DNN’s prediction of patient outcomes to PD-1 immunotherapy [11]. Basically, this technique improves model accuracy by removing the “noisy” genes. First, we used the original DNN trained on the 1093 gene expression data to establish a baseline performance using the accuracy score. Then, we randomly shuffled each gene’s expression values across the 71 testing patients one at a time to disrupt any existing association between that gene and the response classification. After shuffling a gene, the DNN was run again to recalculate the accuracy. If the accuracy decreased after shuffling, that gene was important for predicting the response. Conversely, if the accuracy increased or did not change after shuffling, that gene showed little to no correlation with response prediction. Given the nonlinearity of PD-1 immunotherapy genetics, a standard linear model, such as least absolute shrinkage and selection operator or stepwise regression, is unable to capture the noise in the genes. Feature permutation ignores this weakness by using a direct DNN architecture to quantify the decrease in performance due to a change in the feature. By exploring the performance of the model directly, we remove the uncertainty of a linear model and guarantee the importance of the features in the deployed solution. To evaluate the stability of the features identified, we ran the analysis 3 additional times, each with 50 iterations. We then compared the resulting gene sets to quantify their overlap. We also trained and evaluated the model using each gene set to determine the superior cohort for all subsequent analyses. Equation 2 was used to calculate the importance score assigned to each gene.


(2) $$Importance\;score=accuracy_{baseline}-accuracy_{permutation}$$

#### Training and Testing

We executed our code for the ML models in Google Colab notebooks [44] using an NVIDIA T4 GPU [45] operating with 15 GB of RAM. For all models, 284 patients were used for training, and 71 patients were used for testing. This provided an 80/20 percentage split of the data. For the DNN, an additional validation split of 10% was applied to the training data to monitor model performance during training. This validation set was extracted from the training data, leaving the test set of 71 patients unchanged. During the training of the DNN, an early stopping method was used to monitor the validation loss after each epoch to stop training if the model’s performance diminished. The state of the model was saved after each epoch so that it could revert to the optimal state for testing. This was done to mitigate any overfitting that might occur during training. All ML models were executed 15 times.

#### Evaluation Metrics

To evaluate the models’ performance, we used accuracy, AUC score, recall, specificity, precision, and *F*_1_-scores [46], which are standard metrics used to assess classification performance. These metrics can be found using the confusion matrix, a 2×2 matrix with the number of true positives, true negatives, false positives, and false negatives that the model predicts, with the equations listed below to calculate each metric.


(3) $$Accuracy=\frac{TP+TN}{TP+TN+FP+FN}\;\times \;\in \left[0,1\right]$$

(4) $$Recall=\frac{TP}{TP+FN}\times \in \left[0,1\right]$$

(5) $$Specificity=\frac{TN}{TN+FP}\times \in \left[0,1\right]$$

(6) $$Precision=\frac{TP}{TP+FP}\times \in \left[0,1\right]$$

(7) $$F1=\frac{2\;\times Precision\;\times Recall\;}{Precision+Recall}\;\times \;\in \left[0,1\right]$$

Accuracy (Equation 3) measures the overall correct predictions out of all predictions made. Recall evaluates the model’s ability to correctly identify PD-1 responders as positive out of all PD-1 responders, as shown in Equation 4. Specificity (Equation 5) is the opposite; it measures the model’s ability to correctly identify PD-1 nonresponders out of all nonresponders. Precision (Equation 6) is the ratio of all correctly identified positive PD-1 respondents to all the patients the model assigns as positive, and the *F*_1_-score (Equation 7) is a harmonic mean of precision and recall that penalizes extreme values [47]. AUC measures the trade-off between specificity and recall [38,48].

### Bioinformatics and Statistical Analysis

All computations and analyses in this study were performed in Google Colab notebooks using Python (version 3.10) and R (version 4.4.1). Differentially expressed genes were analyzed with LIMMA in R [30]. Upregulated genes were classified for responders and nonresponders by calculating log fold changes (LogFC). Accuracy, AUC, recall, specificity, precision, *F*_1_-score, true positives, true negatives, false positives, and false negatives were calculated using sklearn Metrics. Statistical analyses were conducted using GraphPad Prism (version 5.01; GraphPad Software). The Kruskal-Wallis nonparametric test, followed by the Dunn post hoc multiple comparison test, was used to compare predictive performance between the models. A *P* value less than .05 was considered statistically significant.

The next section delves into the detailed analysis of the genes identified through the DeepImmunoGene framework and their relevance in predicting immunotherapy response. It outlines how the permutation importance method was used to isolate key genes associated with positive or negative treatment outcomes and discusses the biological significance of these genes in the context of immune response modulation in NSCLC. Additionally, the section provides an in-depth comparison of the ML models’ performance, highlighting the strengths and limitations of each approach, and evaluates their potential applications in clinical settings for improving patient stratification and personalized treatment strategies. By integrating these findings, the study aims to contribute to our understanding of molecular biomarkers that may inform future efforts to optimize the use of PD-1 inhibitors in cancer therapy.

### External Validation

To externally validate the biomarkers identified by DeepImmunoGene, we obtained a bulk RNA-seq dataset (GSE207422) from the GEO public database. This dataset included gene expression data for 58,387 genes across 24 patients with NSCLC who were treated with PD-1 inhibitors combined with chemotherapy [49]. Patient responsiveness was determined using RECIST, where complete response and partial response were considered responders, whereas stable disease was considered a nonresponder. The cohort comprised 17 responders and 7 nonresponders. This external dataset was processed using the aforementioned workflow applied to the training dataset. The Mann-Whitney U test was used to determine whether the difference in gene expression between responders and nonresponders was statistically significant. We generated violin plots of the top-ranked responder and nonresponder biomarkers identified by DeepImmunoGene to assess whether their expression patterns in the test set were consistent with the model’s predictions using the ggplot2 package [50].

### Ethical Considerations

This study used only publicly available or fully deidentified secondary data; therefore, institutional review board approval and informed consent were not required. No personal identifiers were accessed, and privacy and confidentiality were strictly maintained.

## Results

### ML Predicts Response to PD-1 Immunotherapy (RQ1)

**Figure 2.**
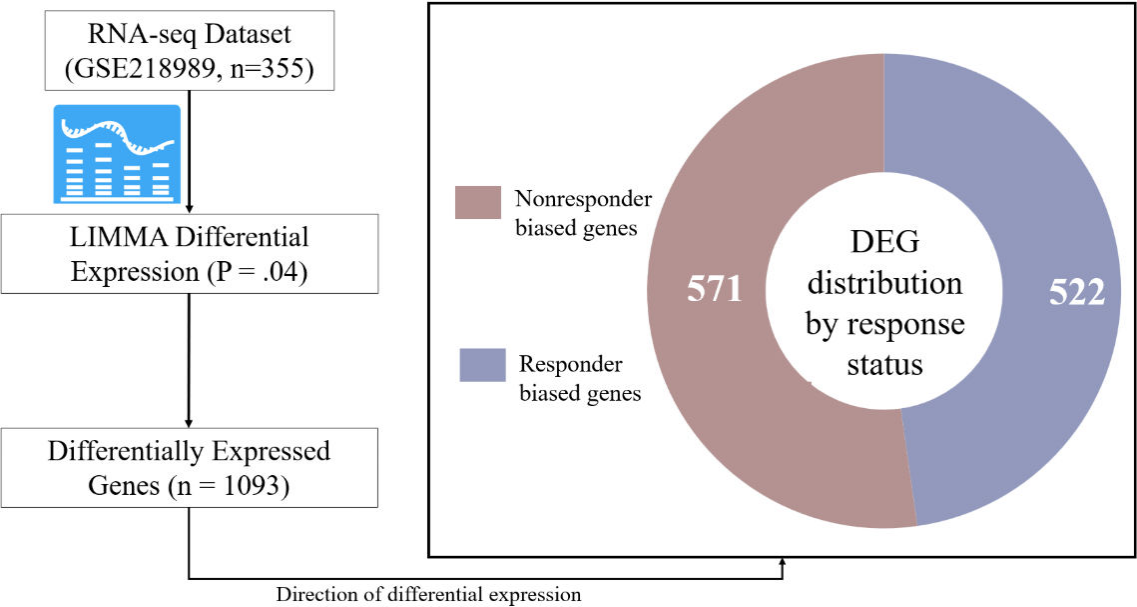
Identification and stratification of differentially expressed genes associated with programmed cell death receptor-1 immunotherapy response in non–small cell lung cancer. Bulk RNA-seq data from 355 patients (GSE218989) were analyzed using LIMMA differential expression analysis (*P*=.04), identifying 1093 differentially expressed genes. These genes were stratified by direction of differential expression into responder-upregulated (n=522) and nonresponder-upregulated (n=571) gene sets, forming the initial feature space for downstream machine learning analyses. DEG: differentially expressed gene.

DEGs were identified using LIMMA power analysis of bulk RNA-seq data (GSE218989) from the GEO public database GEO Repository. LIMMA identified 1093 important DEGs from a total of 19,911 genes in patients with lung cancer, where 522 genes were upregulated in responders, and 571 genes were upregulated in nonresponders (*P*=.04), as shown in Figure 2.

**Figure 3.**
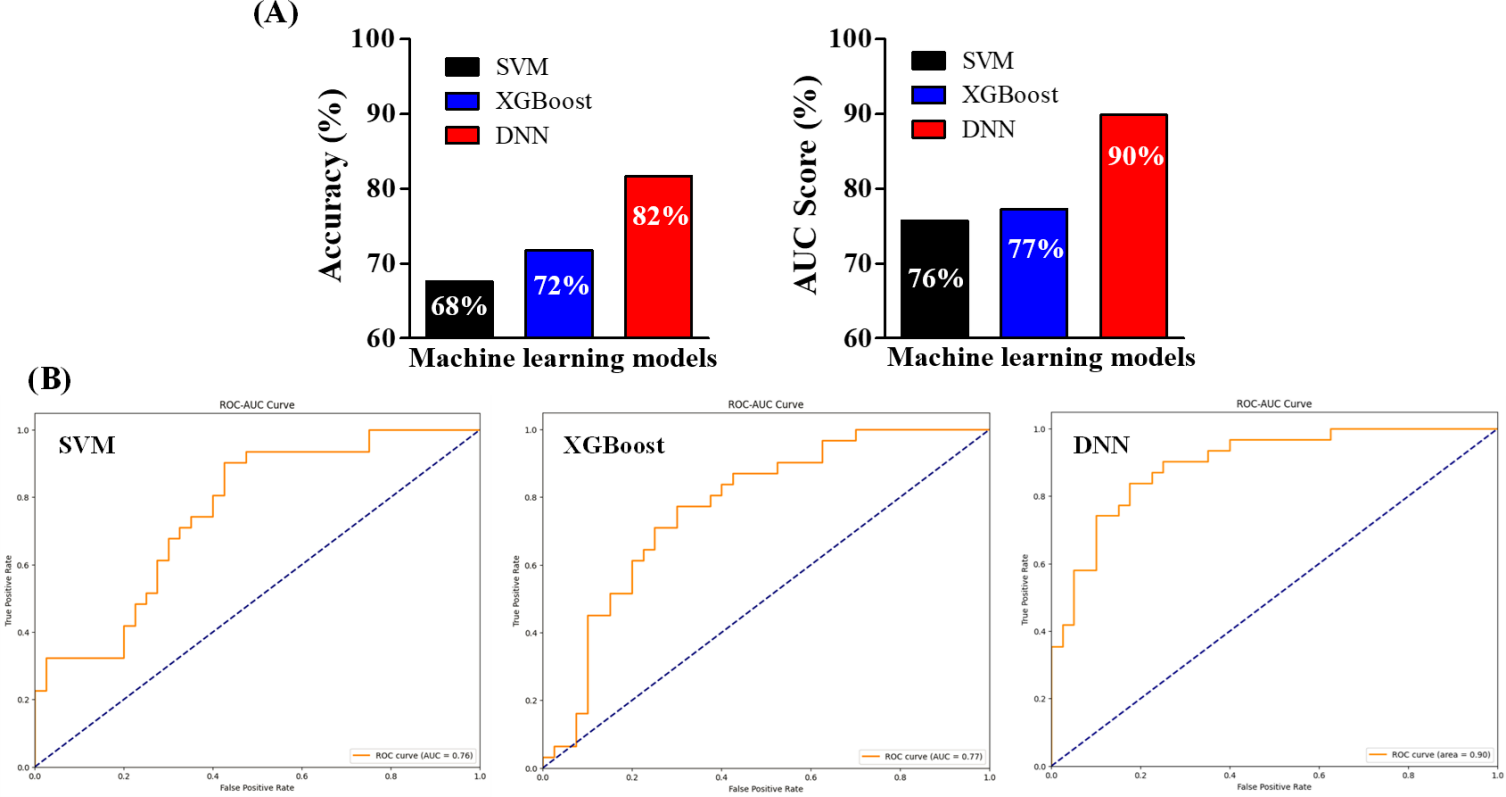
Predictive performance comparison of support vector machine (SVM), extreme gradient boosting (XGBoost), and deep neural network (DNN) models. (A) Accuracy scores and (B) receiver operating characteristic (ROC) curve analysis demonstrate that the DNN model outperformed both SVM and XGBoost. The DNN achieved an accuracy of 82% and an area under the curve (AUC) of 90%, compared to 68% and 76% for SVM and 72% and 77% for XGBoost. These results highlight the advantage of deep learning for modeling complex, high-dimensional gene expression data.

Here, we trained SVM and XGBoost models using the 1093 identified DEGs to predict patient response to PD-1 immunotherapy. The performance of the models was evaluated using several metrics, including accuracy, AUC, recall, specificity, precision, and *F*_1_-score [46]. First, we applied SVM, and our data showed that it achieved an accuracy of 68% and an AUC score of 76% with recall, specificity, precision, and *F*_1_-score values of 0.70, 0.65, 0.77, and 0.71, respectively (Figure 3A, 3B and Table 1). Next, we used XGBoost to see if its ensemble learning method could yield higher accuracy and AUC scores. Our data showed that XGBoost performed slightly better than SVM, with an accuracy of 72%, an AUC score of 77%, a recall of 0.73, a specificity of 0.71, a precision of 0.76, and an *F*_1_-score of 0.74 (Figure 3A, 3B and Table 2). The suboptimal performance of these 2 models may be due to the large dataset, suggesting that a more complex and nonlinear approach, such as a DNN, is necessary for accurately predicting patient responses. We used SVM and XGBoost as baseline classifiers commonly applied in gene expression studies to provide context for the performance of our DNN. While these models differ in complexity from DNNs, the comparison helps demonstrate the added value of capturing nonlinear interactions in gene expression data.

**Table 2.** Performance comparison of machine learning models for predicting response to programmed cell death receptor-1 immunotherapy.

Models	Accuracy	AUC^a^	Recall	Specificity	Precision	*F*_1_-score
SVM^b^ (1093 genes)	0.68	0.76	0.70	0.65	0.77	0.71
XGBoost^c^ (1093 genes)	0.72	0.77	0.73	0.71	0.76	0.74
DNN^d^ (1093 genes)	0.82^e^	0.90^e^	0.85^e^	0.78^e^	0.81	0.84^e^
SVM (98 genes)	0.65	0.75	0.65	0.65	0.70	0.68
XGBoost (98 genes)	0.77	0.81	0.80	0.74	0.80	0.80
DeepImmunoGene (98 genes)	0.87^e^	0.95^e^	0.87^e^	0.89^e^	0.93^e^	0.89^e^

a AUC: area under the receiver operating characteristic curve.

b SVM: support vector machine.

c XGBoost: extreme gradient boosting.

d DNN: deep neural network.

e A statistically significant difference from DeepImmunoGene when compared to SVM or XGBoost.

### DNN Predicts Response to PD-1 Immunotherapy With Higher Accuracy

**Figure 4.**
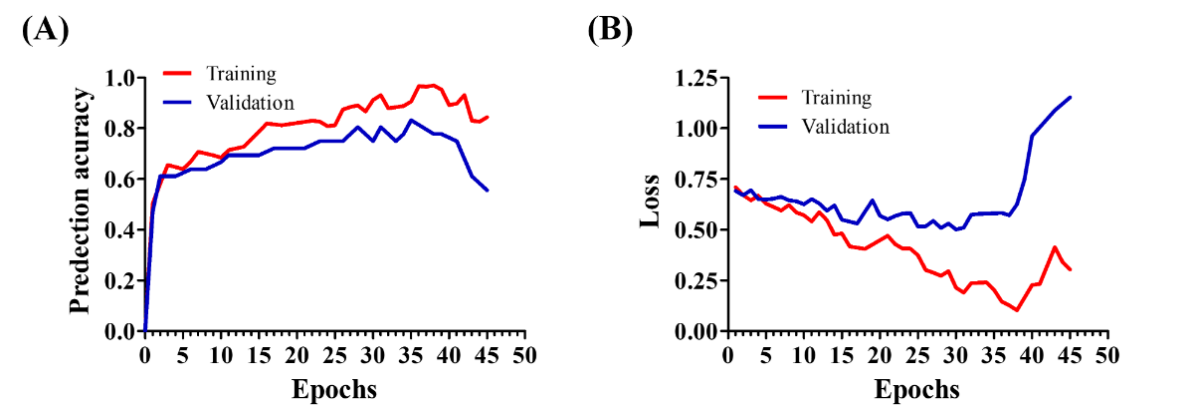
Deep neural network training and validation performance. (A) Training and validation accuracy over epochs shows a steady increase until convergence, with early stopping triggered at epoch 45 and the model reverting to optimal weights from epoch 35. (B) Training loss decreased continuously, whereas validation loss fluctuated slightly before stabilizing, indicating that further training would not significantly improve model performance.

Given that the RNA-seq data includes the expression of more than 1000 genes, we implemented a DNN to enhance predictive accuracy. First, we set the DNN training for 100 epochs, but it stopped at 45 epochs due to early stopping, and the model was then reverted to the optimal state reached at 35 epochs (Figure 4). During the training process, both training and validation accuracy and loss were monitored. We found that the accuracy increased until it exhibited an asymptotic behavior (Figure 4A). Conversely, the training loss decreased steadily, while the validation loss showed some fluctuations (Figure 4B). These findings suggest that training the model for additional epochs would not further improve its performance. Next, we tested the predictive performance. Our data revealed that the DNN achieved excellent predictive performance compared to both SVM and XGBoost, achieving an accuracy of 82%, an AUC score of 90%, a recall of 0.85, a specificity of 0.78, a precision of 0.81, and an *F*_1_-score of 0.84 (Figure 3A, 3B and Table 2). Given the nature of the data, DNN can analyze multidimensional genetic information more accurately than existing linear models. This is showcased with a 21% accuracy improvement over more linear models, such as SVM, and a 14% improvement over XGBoost in our experiments. As a result, we can showcase that to capture the intricacies of the data, it is important to use a model capable of supporting complex multidimensional relationships such as a DNN architecture.

### Key Biomarker Identification (RQ2)

**Figure 5.**
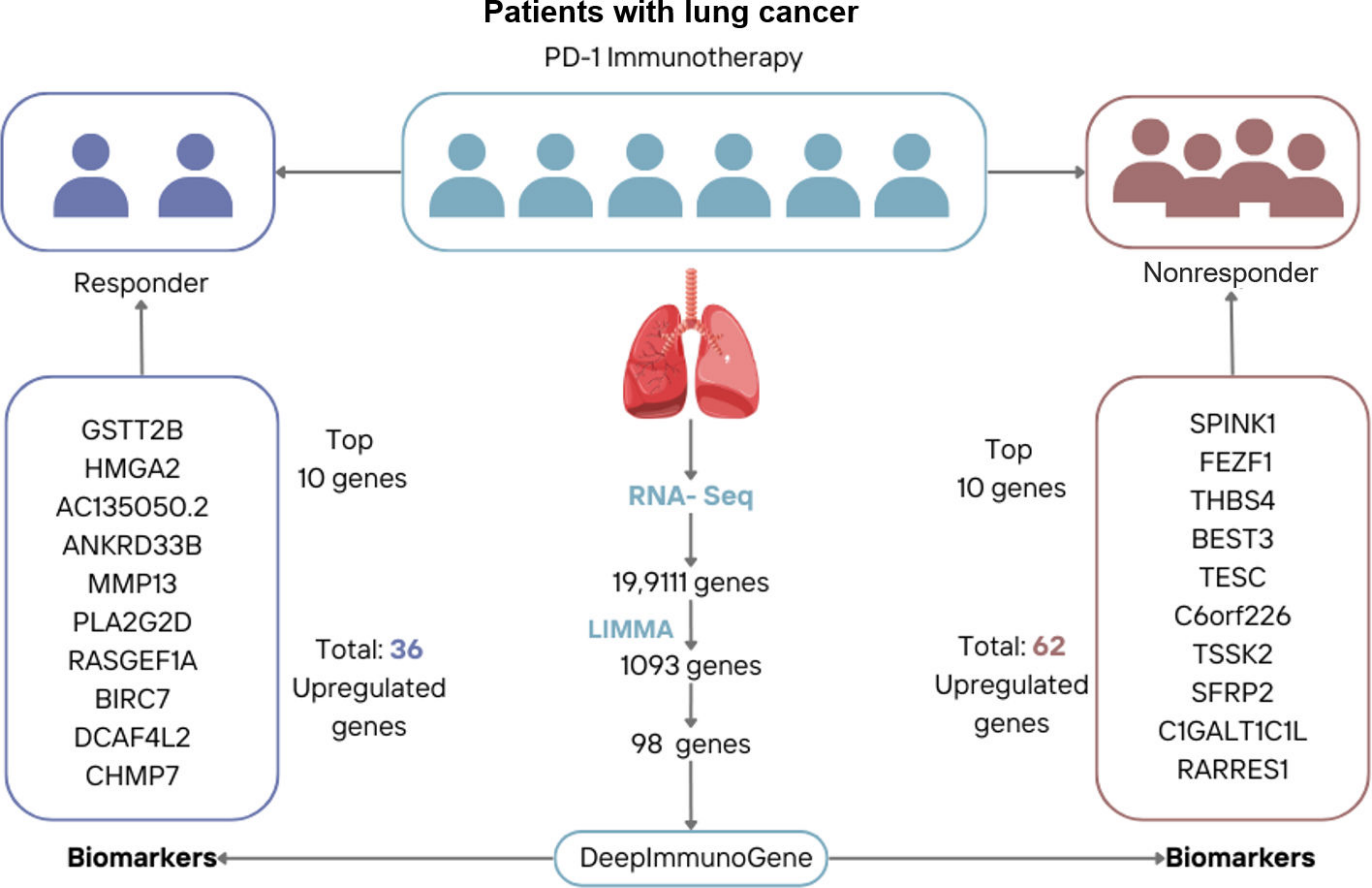
Workflow for identifying predictive biomarkers using DeepImmunoGene. Schematic of the DeepImmunoGene model pipeline. The 1093 differentially expressed genes were subjected to permutation importance analysis to extract the 98 most informative features, which were then used to train the final model. This approach enabled identification of key genes associated with programmed cell death receptor-1 (PD-1) immunotherapy response.

**Figure 6.**
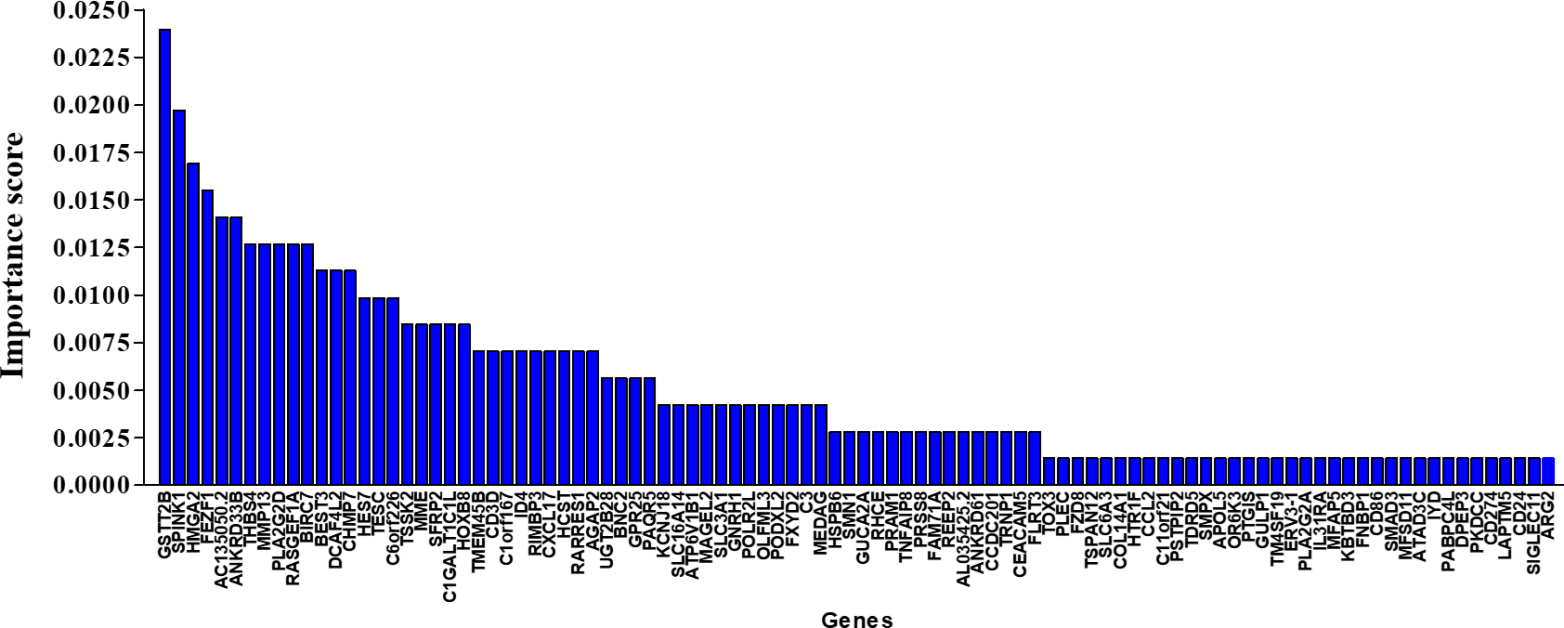
Gene importance ranking using permutation analysis. Permutation importance applied to the 1093 differentially expressed genes using the DeepImmunoGene model identified 98 genes with nonzero importance scores. These genes were ranked based on their contribution to model prediction performance, highlighting their potential as key features for programmed cell death receptor-1 response classification in patients with non–small cell lung cancer.

**Figure 7.**
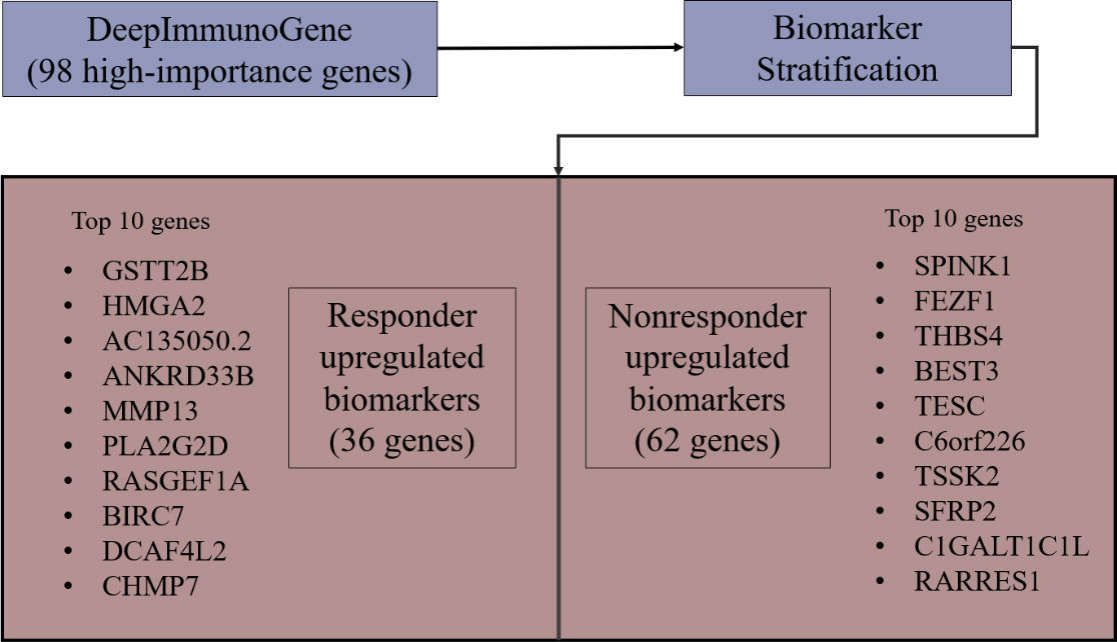
DeepImmunoGene-based stratification of predictive biomarkers associated with programmed cell death receptor-1 (PD-1) immunotherapy response. Using permutation importance and deep neural network modeling, 98 high-importance genes were identified and stratified based on direction of differential expression. Thirty-six genes were upregulated in responders and 62 in nonresponders. The top 10 genes in each group are shown as candidate biomarkers for predicting PD-1 treatment outcomes in non–small cell lung cancer.

We applied DeepImmunoGene with scikit-learn permutation importance to a set of 1093 genes. To mitigate variability in feature importance estimates and to ensure the identification of robust features, this procedure was repeated 3 additional times with 50 iterations each. We then compared the gene sets identified across all 4 total runs and observed a high degree of overlap, with an average of 85.5% consistency among them. The resulting analysis (Figure 5) identified a final set of 98 genes with nonzero importance scores and ranked them according to their level of importance (Figure 6). Although individual gene importance scores below 0.0025 may appear low, the combined contribution of these genes accounts for approximately 18% of the total model importance, indicating they meaningfully improve the model’s predictive performance. These 98 genes were subsequently used to train DeepImmunoGene. Testing this model revealed an accuracy of 0.87 and an AUC of 0.95, a recall of 0.87, a specificity of 0.89, a precision of 0.93, and an *F*_1_-score of 0.89, demonstrating superior performance across all metrics. To validate the necessity of a DL approach for our feature selection and to better contextualize the significant performance improvement of DeepImmunoGene, we conducted a comparative analysis with the traditional ML models. We trained and tested both SVM and XGBoost on the same 98 genes identified via permutation importance. The 98-gene SVM model attained an accuracy of 65%, an AUC of 75%, a recall and specificity of 0.65, a precision of 0.70, and an *F*_1_-score of 0.68. The 98-gene XGBoost model achieved an accuracy of 77%, an AUC of 81%, a recall of 0.80, a specificity of 0.74, a precision of 0.80, and an *F*_1_-score of 0.80 (Table 2). This indicates that DeepImmunoGene outperformed all other models in every metric (Table 2). Genes with a LogFC greater than 0 were considered upregulated in responders, whereas genes with a LogFC less than 0 were considered upregulated in nonresponders. We discovered that 36 genes were upregulated in patients with NSCLC who responded to PD-1 immunotherapy, with the top 10 most significant being GSTT2B, HMGA2, AC135050.2, ANKRD33B, MMP13, PLA2G2D, RASGEF1A, BIRC7, DCAF4L2, and CHMP7 (Figure 7). These genes may serve as potential biomarkers for predicting response to PD-1 immunotherapy. Additionally, we identified 62 upregulated genes in nonresponder patients with NSCLC, with the top 10 most important being SPINK1, FEZF1, THBS4, BEST3, TESC, C6orf226, TSSK2, SFRP2, C1GALT1C1L, and RARRES1 (Figure 7).

The top 10 most significant upregulated genes were identified for both responder and nonresponder patients with NSCLC based on the DeepImmunoGene model. In responders, genes such as GSTT2B, HMGA2, and MMP13 were prominent, whereas SPINK1, FEZF1, and THBS4 were among the top in nonresponders. These genes may serve as potential predictive biomarkers for PD-1 treatment outcomes.

### External Validation of Biomarkers Identified by DeepImmunoGene

**Figure 8.**
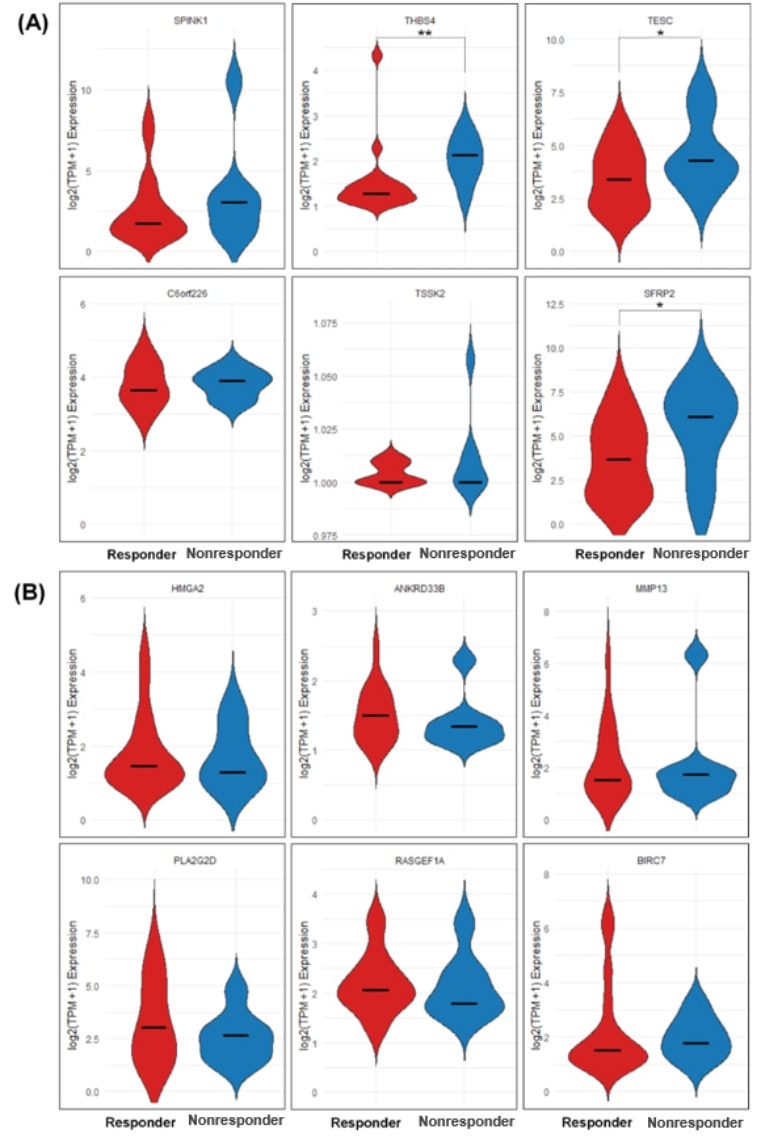
Validation of biomarkers identified by DeepImmunoGene. Violin plots showing differences in the expression of (A) 6 nonresponder-upregulated biomarkers and (B) 6 responder-upregulated biomarkers. *P* values determined by Mann-Whitney *U* test. *P=.05, **P<.01.

Here, we sought to determine whether DeepImmunoGene’s predicted biomarkers showed consistent expression patterns in an independent dataset. We generated violin plots comparing log2 (TPM +1) gene expression between responders and nonresponders. Of the top 10 nonresponder-upregulated biomarkers identified by DeepImmunoGene, 6 genes were present in the independent dataset and analyzed. We found that 4 of these 6 genes (SPINK1, THBS4, TESC, and SFRP2) showed a consistent trend of higher median expression in nonresponders (Figure 8A). Of these, 3 genes (THBS4, TESC, and SFRP2) demonstrated statistically significantly higher expression (*P*=.04) in nonresponders.

Of the top 10 responder-upregulated biomarkers identified, 6 genes were present in the independent dataset and analyzed. We found that 4 of these 6 genes (HMGA2, ANKRD33B, PLA2G2D, and RASGEF1A) showed higher median expression in responders (Figure 8B). BIRC7 and MMP13 had similar median expression in both groups; however, their violin plots displayed extended upper tails, indicating that some patients exhibited markedly higher expression levels. While these patterns suggest differences in expression between responders and nonresponders, statistical significance was not reached in this analysis.

## Discussion

### Principal Findings

We aimed to identify DEGs associated with response to PD-1 immunotherapy in patients with lung cancer using a DNN model to explore the biological mechanisms underlying immunotherapy response. Here, we developed DeepImmunoGene, a computational framework that uses an advanced neural network with an integrated approach to predict patient response to PD-1 immunotherapy with high accuracy. Our model identified 36 upregulated genes, including the top 10 (GSTT2B, HMGA2, AC135050.2, ANKRD33B, MMP13, PLA2G2D, RASGEF1A, BIRC7, DCAF4L2, and CHMP7), which were associated with positive responses to PD-1 immunotherapy in patients with NSCLC. However, apart from the 10 described, our model was able to find approximately 96 total critical genes. If we were to leverage only differential gene expression rather than DeepImmunoGene, more than 1000 genes would be present, many of which are not significant biomarkers for identifying responders. As a result, we deployed a permutation importance feature selector to identify from the potential 1000 expressive genes the ones that are critical in the identification of the patient, reducing the quantity of noisy biomarkers in the dataset. These findings suggest that these genes could serve as the candidate biomarkers for predicting patients who respond to PD-1 inhibitors. Some of these genes (HMGA2, MMP13, BIRC7, and PLA2G2D) have been reported to be overexpressed in various cancers, including lung adenocarcinoma, and are associated with tumor progression and metastasis [51-54], supporting their potential as biomarkers for PD-1 immunotherapy. We can identify these genes by ranking based on feature importance. We identify the most important genes, given the decrease in performance once permutated. The 10 most critical genes show the greatest decline in model accuracy once they are shifted. Furthermore, existing literature has shown many of these genes to be capable identifiers of immunotherapy. Genes such as HMGA2 and MMP13 are currently in the literature to identify a high likelihood of therapy success [55,56]. Our primary contribution lies not in introducing a novel DL architecture, but in developing DeepImmunoGene, a framework that complements prior frameworks, integrating interpretability and ML with the novelty to identify key genomic markers for PD-1 immunotherapy response.

In addition to their differential expression patterns, several of the top-ranked genes identified in our model have established roles in cancer-related biological processes. HMGA2 is a well-characterized architectural transcription factor associated with epithelial-mesenchymal transition and metastatic progression [57]. MMP13 contributes to extracellular matrix degradation and tumor invasion [55]. BIRC7 (also known as Livin) has been implicated in the inhibition of apoptosis and immune evasion mechanisms in solid tumors [58]. PLA2G2D is known for its involvement in inflammatory signaling and has been shown to modulate dendritic cell function and T-cell recruitment in the tumor microenvironment [59]. These functional insights, drawn from existing literature, suggest that many of the identified genes may influence immunotherapy response through diverse oncogenic and immune-related pathways. Although a formal pathway enrichment analysis was not performed, the biological relevance of these genes supports their potential as markers of therapeutic response.

Our analysis began with the application of the LIMMA method to bulk RNA-seq data, which identified 1093 DEGs from a total of 19,911 genes in patients with lung cancer [24]. LIMMA is a widely used tool for differential gene expression analysis, facilitating the identification of genes linked to disease pathogenesis, particularly in RNA-seq and microarray data [30]. We evaluated these 1093 genes using 3 different ML models, including SVM, XGBoost, and DNN, to assess their predictive performance. The SVM showed moderate performance in classifying patient response with an accuracy of 0.68 and an AUC of 0.76, suggesting that it was unable to effectively capture the underlying correlations between gene expression and patient response. This may be due to the nonlinear nature of gene expression data, which likely hindered the SVM model’s ability to generalize its predictions across patients [11,60]. While XGBoost outperformed SVM by a slight margin (0.04 for accuracy and 0.01 for AUC), there is no significant difference between these models, indicating that neither model could provide sufficiently robust predictions. These findings suggest that the high dimensionality, small sample size, and categorical imbalance of RNA-seq data pose significant challenges for traditional ML approaches [61].

To address the limitations of traditional ML models, we applied a DNN, a nonlinear model capable of capturing complex relationships within large gene expression datasets by mimicking the information-processing patterns of the human brain to generate predictions [11,40,60]. Unlike traditional models such as SVM and XGBoost, the DNN consists of multiple layers of neurons connected by weighted links, which allow the model to learn intricate patterns within the data. DNNs have shown strong performance in genomic predictions for various diseases [43]. The DNN model using the 1093 DEGs significantly outperformed both SVM and XGBoost. It exceeded SVM by 14% in both accuracy and AUC and outperformed XGBoost by 10% in accuracy and 13% in AUC. This improved performance of the DNN is attributed to its ability to capture and learn from the high-dimensional, nonlinear interactions inherent in gene expression data, which are challenging for traditional linear models to predict accurately [61]. This capability allows the DNN to generalize more effectively across diverse patient data, leading to more accurate and robust predictions than those made by more basic, linear computational models.

To reduce the number of genes and enhance the reliability of our model, we performed a permutation importance analysis using the scikit-learn framework. This analysis was repeated 4 times, each with 50 iterations to ensure the identification of a robust gene set to build DeepImmunoGene on. This subsequently reduced the set of 1093 genes to 98 genes based on nonzero importance scores, which were correlated with the response to PD-1 inhibitors and ranked according to their importance [62]. The DeepImmunoGene model was then trained using this refined set of 98 genes. Compared to our previous models, DeepImmunoGene demonstrated superior performance and robustness across all metrics (Table 2), indicating that the application of permutation importance effectively eliminated irrelevant, noisy genes, allowing the model to focus exclusively on the most relevant genes without interference during training, such as overfitting. However, we also observed that specificity was consistently slightly lower than recall across all models, indicating that the models had more difficulty discerning nonresponders. This suggests that nonresponders may not have responded to immunotherapy due to external factors, such as the tumor microenvironment, age, or gender [24]. The comparative analysis with traditional ML models using the 98-gene subset found through permutation importance validates the core framework of DeepImmunoGene. The results highlight a specific synergistic effect between our feature selection method and the DNN, which is critical for achieving superior predictive performance. Although reducing the feature set to 98 genes improved computation efficiency no less, the fact that SVM and XGBoost trained on this same reduced feature set still failed to achieve comparable performance suggests that the DNN is better suited to capture the complex, nonlinear relationships and subtle gene-gene interactions underlying the RNA-seq data. Ultimately, the strength of DeepImmunoGene lies in this integrative approach of first identifying the most influential genes for accurate prediction and then leveraging a sophisticated DL model to interpret their combined predictive signal.

Further analysis revealed that 36 genes were upregulated (LogFC>0) in patients who responded to PD-1 immunotherapy, whereas 62 genes were upregulated (LogFC<0) in nonresponders [63]. These results suggest that DeepImmunoGene could serve as a robust ML-based tool for predicting immunotherapy outcomes in patients with lung cancer. The identification of these genes linked to responders and nonresponders not only offers potential biomarkers for predicting immunotherapy success but also enhances our understanding of the molecular mechanisms underlying the immune response in cancer. This could help guide more personalized treatment strategies, ultimately reducing unnecessary side effects and financial burdens for patients and health care systems, as immunotherapy is currently administered without prior knowledge of its effectiveness or safety for each patient [24,26]. Recent studies showed that only approximately 25% of patients show a positive response to immunotherapy, as PD-1/PD-L1 expression is not a sufficient biomarker to select patients who are likely to benefit [25,26]. Therefore, in addition to PD-1/PD-L1 expressions, these genes could be used as clinically actionable biomarkers for predicting response to ICIs with high accuracy.

Finally, we externally validated the predictive biomarkers identified by DeepImmunoGene using an independent bulk RNA-seq dataset of patients with NSCLC treated with PD-1 inhibitors (GSE207422) [49]. Given the small size of the external validation cohort (n=24) and the notable class imbalance (17 responders vs 7 nonresponders), we anticipated limited statistical power to detect meaningful differences (67). Additionally, the dataset itself includes patients receiving PD-1 inhibitors in combination with chemotherapy, which introduces treatment heterogeneity that may cause much of the variations observed in the expression patterns. Despite these limitations inherent to the available data, our analysis found that 4 of 6 nonresponder-upregulated genes showed higher median expression in nonresponders, with 3 achieving statistically significant differences in the predicted direction (*P*<.05). Similarly, 4 of 6 responder-upregulated genes demonstrated higher median expression in responders, although none reached statistical significance. This partial agreement offers encouraging evidence that the model-identified biomarkers capture biologically meaningful expression trends even in an independent, clinically realistic cohort. While these results should be interpreted cautiously, given the small sample size, class imbalance, and treatment variability, they support the potential utility of these gene markers for predicting immunotherapy response. Future validation in larger, well-annotated cohorts with consistent PD-1 treatment protocols is warranted to confirm their clinical relevance, fully validate the model’s predictive classification performance, and further refine the list of biomarkers.

To contextualize DeepImmunoGene among existing approaches, we compared our method to previously published biomarker studies in NSCLC using PD-1 datasets. For example, Hwang et al [64] developed immune gene signatures derived from small patient cohorts with a limited number of features, which can restrict the model’s ability to generalize to diverse patient populations or capture variability in gene expression. In contrast, Ravi et al [65] applied regression-based linear models that assume compounding, independent effects of genes on treatment response, which may fail to capture complex, nonlinear gene-gene interactions. By leveraging a DNN architecture, DeepImmunoGene is designed to learn these nonlinear dependencies across large-scale gene expression data, enabling more comprehensive and potentially generalizable biomarker discovery for predicting immunotherapy response. Other approaches, such as Lee et al [66], propose an ensemble method incorporating different models for the classification from gene expression profiles and additional information. This adds informative features, which may not always be available; in contrast, DeepImmunoGene reduces the feature space of RNA sequencing, helping isolate and detect features that are more likely to carry correct information.

### Conclusions

Our DeepImmunoGene predictive model identified 36 upregulated genes in patients with NSCLC who responded to PD-1 immunotherapy. Among these, the 10 most significant genes (GSTT2B, HMGA2, AC135050.2, ANKRD33B, MMP13, PLA2G2D, RASGEF1A, BIRC7, DCAF4L2, and CHMP7) may serve as potential genomic biomarkers for predicting which patients with NSCLC are most likely to respond to PD-1 immunotherapy. Our external validation on an independent cohort supported several of the model-identified biomarkers, demonstrating partial agreement with DeepImmunoGene’s predicted expression patterns despite the small sample size and class imbalance. These findings offer a promising foundation for future research aiming to improve patient stratification for PD-1 immunotherapy. Further validation in larger, well-annotated datasets and biological systems is needed to confirm their correlation with PD-1 inhibitors, which could lead to the development of more personalized and effective immunotherapies for lung cancer. Although the DeepImmunoGene model demonstrated promising predictive performance, this study has several limitations. First, the analysis was conducted on a relatively small cohort of 355 patients with lung cancer. Second, we relied on a single publicly available RNA-seq dataset, which limited our ability to perform external validation. Third, key demographic and clinical variables, such as cancer stage, NSCLC subtype, age, and sex, were not available in the dataset. These factors are known to influence both immune response and gene expression, and their absence restricts the model’s robustness assessment across patient subgroups. As a result, we were unable to evaluate the potential influence of demographic biases on model predictions. Future work with more comprehensive and diverse datasets is essential to validate the model’s generalizability and to assess its consistency across clinically relevant subpopulations. We plan to conduct a follow-up study using external datasets when available and collaborate with clinics to validate our findings and further refine the list of biomarkers.

We also acknowledge that more advanced DL models exist for this task. Future work will involve evaluating DeepImmunoGene against state-of-the-art architectures, incorporating multimodal data, and validating performance on larger and more diverse cohorts. In this study, while DeepImmunoGene demonstrated strong performance metrics, future research should focus on improving the model’s robustness through external validation across diverse datasets, including those from different geographical regions, patient demographics, and cancer stages. This would help assess how well the model generalizes beyond the current cohort of 355 patients. Moreover, the bias-variance tradeoff is crucial in this context. Our current model, which is highly sophisticated (DNN), likely strikes a balance between bias and variance, but there may still be room for improvement. High bias could occur if the model is overly simplified, missing important patterns in the data, whereas high variance could result from overfitting the model to the training data, leading to poor performance on new, unseen data.

## Abbreviations

AUC: area under the receiver operating characteristics curve
DEG: differentially expressed gene
DL: deep learning
DNN: deep neural network
GEO: Gene Expression Omnibus
ICI: immune checkpoint inhibitor
LogFC: log fold changes
ML: machine learning
NSCLC: non–small cell lung cancer
PD-1: programmed cell death receptor-1
PD-L1: programmed cell death-ligand 1
RECIST: Response Evaluation Criteria in Solid Tumors
RQ: research question
SCLC: small cell lung cancer
SVM: support vector machine
TPM: transcripts per million
XGBoost: extreme gradient boosting
